# Glutathione in Cerebral Microvascular Endothelial Biology and Pathobiology: Implications for Brain Homeostasis

**DOI:** 10.1155/2012/434971

**Published:** 2012-06-17

**Authors:** Wei Li, Carmina Busu, Magdalena L. Circu, Tak Yee Aw

**Affiliations:** Department of Molecular and Cellular Physiology, Louisiana State University Health Sciences Center, Shreveport, LA 71130-3932, USA

## Abstract

The integrity of the vascular endothelium of the blood-brain barrier (BBB) is central to cerebrovascular homeostasis. Given the function of the BBB as a physical and metabolic barrier that buffers the systemic environment, oxidative damage to the endothelial monolayer will have significant deleterious impact on the metabolic, immunological, and neurological functions of the brain. Glutathione (GSH) is a ubiquitous major thiol within mammalian cells that plays important roles in antioxidant defense, oxidation-reduction reactions in metabolic pathways, and redox signaling. The existence of distinct GSH pools within the subcellular organelles supports an elegant mode for independent redox regulation of metabolic processes, including those that control cell fate. GSH-dependent homeostatic control of neurovascular function is relatively unexplored. Significantly, GSH regulation of two aspects of endothelial function is paramount to barrier preservation, namely, GSH protection against oxidative endothelial cell injury and GSH control of postdamage cell proliferation in endothelial repair and/or wound healing. This paper highlights our current insights and hypotheses into the role of GSH in cerebral microvascular biology and pathobiology with special focus on endothelial GSH and vascular integrity, oxidative disruption of endothelial barrier function, GSH regulation of endothelial cell proliferation, and the pathological implications of GSH disruption in oxidative stress-associated neurovascular disorders, such as diabetes and stroke.

## 1. Glutathione and Neurovascular Homeostasis

### 1.1. Function of the Blood-Brain Barrier

Central to neurovascular homeostasis is the function of the blood-brain barrier (BBB). The BBB is a highly regulated interface between the systemic circulation and brain parenchyma and is comprised of a monolayer of brain capillary endothelial cells on the blood side and perivascular cells on the brain side of microvessels. The BBB functions to protect the parenchymal cells from fluctuations in plasma composition, such as during exercise and following meals, and against circulating neurotransmitters or xenobiotics capable of disrupting neural function [1]. In this regard, the BBB acts as a mechanical barrier; brain capillaries are ~50–100 times tighter than peripheral microvessels, a property that is attributed to intercellular tight junctions between neighboring endothelial cells that restrict the paracellular diffusion of hydrophilic solutes. Only small molecules such as oxygen and CO_2_ can freely diffuse across the lipid membranes of the endothelium.

On the luminal and abluminal membranes, specific transport systems regulate the transcellular traffic of small hydrophilic molecules, such as GLUT-1 and L-system carrier 1 in the transport of glucose or leucine, respectively, thereby providing a selective “transport barrier” that facilitates nutrient entry [2]. The highly expressed P-glycoprotein transporter on endothelial luminal surface protects the brain from xenobiotics and the potentially toxic neurometabolite, glutamate. In addition, an enrichment of endothelial degradative enzymes serves as an enzymatic barrier. Examples include ectoenzymes such as peptidases and nucleotidases, which metabolize peptides and ATP, respectively, and the intracellular enzymes monoamine oxidase and cytochrome P450 1A and 2B which inactivate blood-borne neuroactive compounds. Moreover, the cerebral endothelium exhibits specific systems for receptor-mediated and adsorptive endocytosis that allow for the transfer of specific peptides and lipoproteins to the brain [2]. Such multiple functions of the BBB regulate the brain microenvironment and maintain parenchymal homeostasis.

### 1.2. Glutathione Redox System and Cellular Function

The glutathione/glutathione disulfide (GSH/GSSG) couple is the most abundant thiol redox system that plays a key role in the maintenance of the redox environment in cells [3, 4]. Under physiological conditions, intracellular GSH homeostasis depends on *de novo *GSH synthesis, GSH redox cycling, and transmembrane GSH transport. Cellular GSH exists mainly in the reduced form with GSSG constituting less than 10% of the total GSH pool. The biological functions of GSH are attributed to its unique *γ*-glutamyl bond between the glutamate and cysteine residues and to the presence of a free thiol group. Reduced GSH is synthesized in the cytosol in two steps from its constituent amino acids (glutamate, cysteine, glycine) catalyzed by *γ*-glutamyl cysteine ligase (GCL) and GSH synthase [5]. GCL catalyzes the formation of *γ*-glutamylcysteine, the first and rate-limiting reaction in GSH synthesis, and enzyme function is controlled by GSH feedback inhibition or by transcriptional upregulation of enzyme subunits (Section 1.4). An important aspect of cellular GSH homeostasis is that increased GSH oxidation is generally followed by increases in the total pool size, notably through enhanced *de novo *GSH synthesis.

The versatility of GSH in contributing to a myriad of cellular functions is notable in its role in detoxication reactions (e.g., hydroperoxide and xenobiotic catabolism), regulation of amino acid transport into cells, maintenance of native three-dimensional protein structure in biosynthetic/metabolic processes (e.g., prostaglandins D2 and E2 synthesis), serving as a cofactor for enzyme systems (e.g., glyoxalase I), and redox signaling. Thiol-disulfide exchanges and protein S-glutathiolation are mechanisms by which GSH modulates the oxidative modification of redox active cysteines within proteins and thereby regulates the activity of a variety of enzyme functions, including those controlling proliferation, differentiation, or apoptosis [6, 7].

#### 1.2.1. Subcellular Distribution of GSH

Intracellular GSH is differentially distributed among the various subcellular compartments of cytosol, mitochondria, endoplasmic reticulum, and nucleus wherein distinct redox pools are formed [8, 9]. Cytosolic GSH is highly reduced, and under physiological conditions cytosolic GSH concentrations are between 1 and 11 mM with the GSH to GSSG ratio maintained in excess of 10 to 1 depending on cell types [10]. The redox state of a cell is generally represented by the ratio of GSH to GSSG given the large GSH pool size. Quantitatively, the cytosolic pool accounts for >70% of the total cellular GSH, while the nuclear and mitochondrial compartments comprise 10% to 30% of the total cellular GSH, respectively [11]. The uniqueness of the nuclear and mitochondrial GSH pools is evidenced by the differences in compartmental GSH turnover rate and sensitivity to chemical depletion [9]. Specifically, the distinct characteristic of the nuclear GSH redox state is consistent with its physiological role in the nucleus, significantly during cell cycle [12] (Section 3.2 below). Indeed, increased nuclear-to-cytosol GSH distribution is a crucial factor in cell proliferation wherein elevated nuclear GSH maintains the functional integrity of the nucleus in gene transcription [13].

While the biological importance of metabolically unique GSH compartments in redox regulation of various endothelial cell functions [14, 15] is yet to be fully defined, it can be readily appreciated that such independent GSH pools would afford an elegant mechanism for specific control of redox-sensitive metabolic processes, the failure of which will have significant implications for endothelial pathobiology. The reader is referred to previous excellent reviews for a full discussion of redox compartmentation and its integration in redox signaling [3, 8, 15].

#### 1.2.2. GSH in Cellular ROS and Redox Signaling

One of the undesired consequences for an organism living in an aerobic environment is an increased potential for oxidative damage by reactive oxygen species (ROS). However, the ability to thrive within such an aerobic environment also implies an evolved capability to handle ROS-mediated tissue damage [16]. The major intracellular sources of ROS, namely, superoxide anion (O_2_^∙−^), hydrogen peroxide (H_2_O_2_), or hydroxyl radical (HO^∙^), are derived from mitochondrial respiration, arachidonic acid pathway, and activities of cellular oxidases, such as cytochrome P450, glucose oxidase, amino acid oxidases, xanthine oxidase, NADH/NADPH oxidases, or NO synthases [17, 18]. ROS derived from xenobiotic metabolism or UV/*γ*-radiation are examples of exogenous sources. Elevated ROS levels are damaging to cellular macromolecules like proteins, lipids, and DNA and will induce a state of oxidative stress and redox imbalance [8]. Central to maintaining intracellular redox balance is GSH-dependent ROS elimination that includes GSH peroxidase-catalyzed hydroperoxide metabolism, GSSG reductase-catalyzed, NADPH-dependent GSH regeneration, or GSH S-transferase-catalyzed xenobiotic detoxication [19].

The recognition that ROS can serve as important mediators of cell signaling and that signal transduction may be mediated by ROS-induced GSH redox imbalance is major conceptual breakthrough in our understanding of GSH-dependent redox signaling [20, 21]. Significantly, low ROS levels participate in the signaling of proliferation, senescence, and apoptosis. For instance, H_2_O_2_-targeted proteins containing redox sensitive cysteine residues (P-SH) can result in the formation of reversible sulfenic (P-SOH) as well as irreversible sulfinic (P-SO_2_H), and sulfonic (P-SO_3_H) acid derivatives [22, 23]. The protein sulfenic acid derivative can further react with nitric oxide (NO) to yield nitrosothiol (P-SNO) or with another P-SH to form a disulfide bond (P-SS-P) [22, 23]. The latter posttranslational modification, termed S-glutathiolation (also known as S-glutathiolation), refers to the formation of a mixed disulfide between the cysteine of GSH and a cysteine moiety of a protein [6]. Reversible protein cysteine oxidation and protein mixed disulfide formation are catalyzed by the thioredoxin (Trx) and glutaredoxin (Grx) family of redox proteins [6]. This GSH-protein cysteine interaction protects against irreversible protein thiol oxidation and is an important redox mechanism in regulating protein function at low or modest levels of ROS [6]. ROS-dependent protein cysteine oxidation has been implicated in the redox regulation of a wide range of protein functions including enzyme activity, protein expression and abundance, subcellular protein localization, and interaction with other molecular partners in controlling new patterns of cell signaling and gene expression. Viewed simply, control of protein functions by reversible S-glutathiolation/deglutathiolation is akin to that of phosphorylation/dephosphorylation.

### 1.3. Endothelial GSH and S-Glutathiolation in the Control of Vascular Integrity

**Figure 1:**
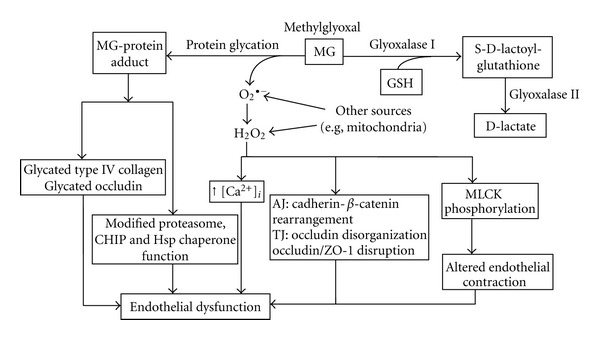
Mechanisms of MG-mediated endothelial barrier dysfunction and its protection by GSH. MG-induced endothelial barrier dysfunction can be caused by MG-protein crosslinking (glycation) resulting in the formation of MG-protein adducts, such as tight junction occludin and basement membrane type IV collagen. MG-protein glycation can also modify the proteasomal and chaperone functions. ROS generated during protein glycation can further mediate barrier dysfunction through various pathways: (a) increased intracellular [Ca^2+^], (b) direct disruption of adherens junction and tight junction, or (c) phosphorylation of myosin light chain kinase and altered endothelial cell contraction. Protection of barrier integrity is mediated by GSH, which functions as a cofactor in glyoxalase I-catalyzed metabolism of MG. MG: methylglyoxal, GSH: reduced glutathione, O_2_^∙−^: superoxide anion, H_2_O_2_: hydrogen peroxide, ROS: reactive oxygen species, AJ: adherens junction, TJ: tight junction, MLCK: myosin light chain kinase.

GSH exerts profound effects on vascular endothelial function, which include endothelial barrier permeability [24], cell apoptosis [25], chemotaxis, angiogenesis [26, 27], constitutive and agonist-induced adhesion molecule expression [28], leukocyte-endothelial adhesion response [29], and endothelial dependent vasodilation [28, 30]. The modulatory effects of GSH are accomplished through the scavenging of ROS [31], an important second messenger in many endothelial functions. For instance, GSH was shown to attenuate H_2_O_2_-induced decrease in transendothelial electrical resistance via negative regulation of the activation of p38 MAP kinase [24]. In other roles, reduced GSH acts as a substrate for the detoxication enzymes, GSH peroxidase, and GSH S-transferase. Our recent studies showed that GSH served as a cofactor in glyoxalase 1-catalyzed detoxication of methylglyoxal and prevented carbonyl stress-induced brain endothelial barrier dysfunction (Figure 1).

 A large body of evidence supports a role for S-glutathiolation in redox regulation of vascular function, ranging from cell signaling, apoptosis, protein folding, to cytoskeletal reorganization. In hypertensive vessels, the thiolation of endothelial nitric oxide synthase (eNOS) is pivotal in the redox control of vascular tone. The bioactive nitric oxide (NO) molecule plays a crucial role in normal endothelial function, including modulation of vascular dilator tone, inhibition of platelet activation, inhibition of leukocyte adhesion and migration, and inhibition of smooth muscle cell migration and proliferation [32]. Therefore, altered NO production, such as during oxidative stress, would compromise vascular homeostasis. Oxidative stress has been shown to mediate S-glutathiolation of eNOS that was associated with decreased NOS activity, attenuated NO production, increased O_2_^∙−^ generation, and impaired endothelium-dependent vasodilation, dysregulated processes that were restored by thiol-specific reducing agents [33]. As for cell signaling, oxidants have been shown to trigger direct S-glutathiolation of p21ras at Cys^118^, which activated p21ras and mediated downstream phosphorylation of ERK and AKT in both endothelial and smooth muscle cells [34, 35]. Similarly, oxidant-induced insulin resistance was mediated through S-glutathiolation of p21ras and ERK-dependent inhibition of insulin signaling [36]. During diamide-induced oxidative stress, activation of endothelial Ca^2+^ signaling was associated with S-glutathiolation of the inositol-1,4,5-trisphosphate (IP3) receptor (IP3R) and the plasmalemmal Ca^2+^-ATPase pump, which promoted Ca^2+^ release from IP3-sensitive internal Ca^2+^ stores and elevated basal [Ca^2+^]i in the absence of extracellular Ca^2+^ [37].

 Current evidence implicates the involvement of S-glutathiolation/deglutathiolation in apoptotic signaling. In TNF-*α*-mediated apoptosis, Grx-catalyzed deglutathiolation of procaspase-3 induced caspase-3 activation [38]. In Fas-mediated apoptosis, Fas thiolation following caspase-dependent Grx1 degradation resulted in the activation of caspases-8 and -3 [39]. Molecular chaperones are an interesting class of proteins that are readily S-glutathiolated wherein thiolated proteins exhibited potentiation of chaperone activities, such as the correct folding of newly synthesized polypeptides [6]. The activities of several S-glutathiolated members of the glucose-related protein (GRP) family of proteins including GRP78, heat shock protein 60 (Hsp60), heat shock cognate 71-kDa protein, and Hsp90 were similarly increased by S-glutathiolation in diamide-treated endothelial cells [40]. Remarkably, even endothelial cytoskeletal reorganization can be modulated by protein S-glutathiolation, notably that of actin and tubulin. Under physiological conditions, S-glutathiolated actin (at Cys^374^) inhibited F-actin polymerization, which was reversed by EGF via actin deglutathiolation [41], consistent with a dynamic role of actin assembly/disassembly in the biological process of cell division and cell growth. Notably, actin-glutathiolation also occurred under conditions of oxidative stress [42]; in this instance, intracellular actin disassembly or disrupted actin-junctional protein interactions would likely mediate the loss of endothelial barrier function. A role for S-glutathiolated annexin A2-actin interaction is currently unknown. Similarly, while S-glutathiolation of endothelial *β*-tubulin has been reported [40], the biological importance of this modification in endothelial barrier function remains to be defined.

### 1.4. GSH Regulation: Transcriptional Control of GCLc and GCLm Expression

 GCL-catalyzed *de novo *synthesis is central to the preservation of tissue GSH balance, particularly during oxidative stress. GCL is a heterodimeric protein composed of catalytic (GCLc) and modifier (GCLm) subunits. The GCLc subunit alone possesses all of the catalytic activities of the enzyme; however, heterodimerization with the GCLm subunit increases GCL activity (*V*_*max*_ and *K*_cat_), substrate affinity (*K*_*m*_) for glutamate and ATP, and the *K*_*i*_ for GSH feedback inhibition [43]. Metabolic regulation of GCL is mediated by protein phosphorylation at serine and threonine moieties, which inhibits enzyme activity and transcriptional control of GCL function is through the expression of the catalytic and modulatory subunits.

#### 1.4.1. Regulation of GCL Catalytic (GCLc) and Modifier (GCLm) Subunits

The promoters of GCLc and GCLm subunits share common elements and coordinate transactivation results in overall increase in subunit expression. Key mediators of GCL expression are the redox sensitive transcription factors, nuclear factor kappa B (NF-*κ*B), Sp-1, activator protein-1 and -2 (AP-1, AP-2), and nuclear factor E2-related factor 2 (Nrf2) [44]. The promoter of the human *GCLc* gene contains consensus binding sites for AP-1, NF-*κ*B, Nrf2, and for the antioxidant response (ARE) or electrophile responsive (EpRE) elements [44]. A proximal AP-1 element was crucial for the transcription of GCLc induced by oxidative stress [45] while NF-*κ*B was essential in TNF*α*-mediated increase in GCLc transcription either directly or indirectly via transactivation of AP-1 sites through induction of C-Jun expression [43]. Among the four AREs in the human GCLc promoter, ARE4 was important in the constitutive expression of hepatic GCLc induced by *β*-naphthoflavone (*β*-NF) or cytochrome P450 2E1 [46, 47]. In macrophages, elevated GCLc expression caused by homocysteine was mediated by ARE4 and the MERK-ERK1/2 kinase pathway [48]. Involvement of the PI3 kinase pathway was also described in adrenomedullin-induced transcriptional activation of the GCLc promoter [49]. Recent studies from our laboratory demonstrated a role for Nrf2 in the constitutive and insulin-induced endothelial GCLc expression [50]. Insulin-induced GCLc promoter activation was ARE4 dependent [51]. Significantly, the increase in GCL activity and GSH synthesis via insulin signaling and activation of the PI3K/Akt/mTOR/Nrf2/GCLc pathway prevented hyperglycemia-induced endothelial apoptosis [52]. Interestingly, rat GCLc promoter exhibited only three AREs in the 5′-flanking region, of which ARE3 was involved in Nrf2-dependent expression of GCLc [53], suggesting species differences in ARE requirements for GCLc activation.

 Constitutive or induced posttranslational phosphorylation of GCLc further contributes to GCL control. In contrast to insulin and hydrocortisone, which induced GCLc gene expression [17], stress hormones such as glucagon and phenylephrine caused GCLc phosphorylation through activating the protein kinases, PKA, PKC, or Ca^2+^-calmodulin kinase [54, 55]. Notably, GCLc phosphorylation decreased GCLc activity.

 The transcriptional regulation of GCLm is poorly understood. Current evidence shows that the human GCLm promoter also contained an ARE site that mediated Nrf2-dependent GCLm upregulation induced by *β*-NF and lipid peroxidation products [56, 57]. In rat liver, an ARE element similarly mediated the basal and TNF*α*-induced of the GCLm promoter activity [58]. Additionally, the rat GCLm promoter has an AP-1 consensus site for constitutive and tert-butylhydroquinone-induced GCLm expression. For reasons yet unclear, NF*κ*B-dependent GCLm expression appeared to be linked to AP-1 activation within the GCLc promoter [43], suggesting possible cross-talk between the two promoters in subunit expression.

## 2. Oxidative Challenge and Endothelial Barrier Dysfunction

### 2.1. Influence of Reactive Oxygen Species (ROS)

 It is abundantly clear that oxidative stress induced by ROS such as O_2_^∙−^, HO^∙^, or H_2_O_2_ can elicit endothelial barrier dysfunction. Moreover, oxidative stress also increased intracellular endothelial calcium concentration ([Ca^2+^]i) [59, 60]; in pulmonary artery endothelial cells, the blockade of Ca^2+^ entry abolished oxidative stress-induced solute permeability [61], indicating that oxidative stress was linked to elevated [Ca^2+^]i, an important modulator of endothelial permeability (Figure 1). In addition, oxidants like H_2_O_2_ were shown to increase the phosphorylation of myosin light chain kinase [62], suggesting that ROS can alter endothelial contraction and contribute to endothelial barrier dysfunction (Figure 1). This means that oxidant modulation of the cytoskeletal architecture of the endothelial monolayer could be central to the loss of barrier integrity. Moreover, increased ROS concentrations can decrease NO bioavailability through chemical inactivation to form the powerful oxidizing agent, peroxynitrite [63]. Tetrahydrobiopterin (BH4), a critical cofactor for eNOS function, is a crucial target for oxidation by peroxynitrite [64]. Significantly, BH4 oxidation and depletion were shown to induce eNOS uncoupling, a process that was associated with increased O_2_^∙−^ generation and decreased NO production. In this regard, uncoupled eNOS is akin to a dysfunctional O_2_^∙−^ generating enzyme that could contribute to endothelial oxidative stress and vascular dysfunction. The uncoupling of eNOS has been demonstrated *in vitro* and in hypertensive rat (SHR) models of cardiovascular pathophysiology, such as angiotensin-II-induced hypertension and diabetes [65].

 Control of paracellular permeability in the endothelium is a function of the intercellular endothelial adherens junctions (AJ) and tight junctions (TJ), a complex structure comprised of specific junctional proteins. The cadherins, *α*-catenin, and *β*-catenin proteins are components of the AJ, while the transmembrane proteins, occludin, claudin, junction adhesion molecule, and the cytoplasmic accessory zonula occludin (ZO-1, -2, and -3) proteins comprised the TJ [66]. H_2_O_2_-induced barrier disruption has been shown to occur through rearrangement of endothelial cadherin and *β*-catenin and the disruption of *β*-catenin/cytoskeletal association [67], but the signaling events are unresolved. However, activation of ERK1/ERK2 signaling and occludin phosphorylation were shown to mediate the disorganization of occludin and the disruption of occludin-ZO-1 interactions on endothelial cell surfaces [68]. ROS activation of signaling pathways, such as PKC, may further regulate the phosphorylation state of other AJ and TJ proteins. In this regard, a reversal of thrombin-induced loss of the cadherin junctional proteins, *ρ*-catenin, *α*-catenin, and p120, by PKC inhibitor has been described [69].

### 2.2. Role of Carbonyl Stress

 Carbonyl stress is the result of enhanced reactive carbonyl species (RCS) production and decreased carbonyl-scavenging capability, leading to tissue accumulation of reactive dicarbonyl species, such as methylglyoxal (MG). MG is produced from cellular glycolytic intermediates and can induce carbonyl stress through irreversible reaction with free arginine residues of proteins to form advanced MG-glycated end product (AGE) [70] (Figure 1). The generation of protein carbonyls or protein-glycated products could be a major problem in diabetic neurovascular pathology. An MG-derived argpyrimidine adduct has been detected in human lens and kidney and in atherosclerotic lesions of diabetic patients [71–73], and argpyrimidine-modified heat shock protein 27 (Hsp 27) was shown to alter diabetic endothelial cell function [74]. Moreover, diabetes-associated hyperglycemia and MG-induced modification of the corepressor *mSin3A *gene were linked to elevated angiopoietin-2 transcription in microvascular endothelial cells [75]. Other evidence revealed that the modification of 20S proteasome by MG decreased proteasomal chymotrypsin-like activity and impaired the CHIP and chaperone-dependent quality control of the protein [76], leading to the accumulation of toxic aggregates and endothelial cell death. Further, MG-induced glycation of vascular basement membrane type IV collagen yielded hotspots of arginine-derived hydroimidazolone residues at RGD and GFOGER integrin-binding sites, causing endothelial cell detachment, anoikis, and inhibition of angiogenesis [77].

 The crosslinking of MG and amino acids was shown to yield the O_2_^∙−^ radical anion [78] that can be quenched by O_2_^∙−^ scavenger and membrane-permeable catalase [79]. Significantly, MG-derived ROS has important implications for vascular and endothelial function. It is noteworthy that MG-induced mitochondrial O_2_^∙−^  generation stimulated eNOS activity [79], while MG-mediated eNOS phosphorylation (at ser^1777^) attenuated endothelial NO production [80], suggesting that carbonyl stress modulation of endogenous endothelial NO production is a complex process. In rat carotid arterial endothelium MG was found to augment AT1R-induced NADPH oxidase-derived mediated O_2_^∙−^ and H_2_O_2_ production, which increased Ang II-dependent vascular contraction [81]. Similarly, MG-derived ROS mediated the oxidative and hyperglycemic stress-induced impairment of endothelium-dependent vasorelaxation. This oxidative stress response was attenuated by the overexpression of glyoxalase I which promoted MG degradation [82]. In recent studies, we found that MG-occludin glycation induced barrier dysfunction of human brain microvascular endothelial cells; surprisingly, MG-dependent endogenous ROS generation did not contribute majorly to barrier dysfunction. Our study further revealed that the endothelial GSH status is a determinant of barrier integrity by facilitating glyoxalase I-catalyzed MG metabolism and thereby decreasing the availability of free MG (Li and Aw, unpublished data, Figure 1). Moreover, GSH depletion significantly promoted MG-induced endothelial oxidative stress and cell apoptosis [25, 83].

 Altered cell morphology, aberrant cytoskeletal rearrangement, and ZO-1 loss were notable biological consequences of glyoxal, another sugar-derived aldehyde product. Additionally, glyoxal also elicited mitochondrial dysfunction, inhibition of DNA and cell replication, and cell cytotoxicity through protein carbonyl formation [84]. Collectively, these findings underscore the wide-ranging cellular effects of carbonyl stress on vascular endothelial function.

## 3. Endothelial Repair through Proliferation and Growth

### 3.1. Biology of Cell Cycle Control

**Figure 2:**
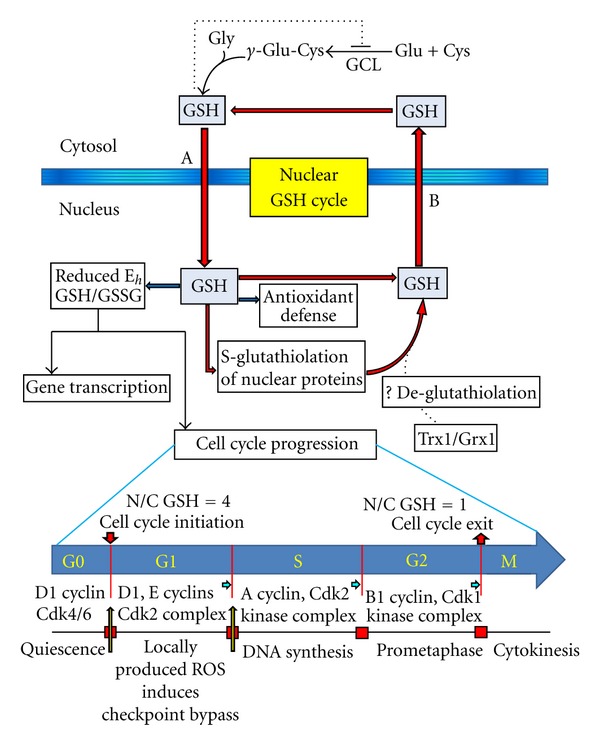
Nuclear glutathione cycle and associated redox changes during cell cycle progression. A nuclear GSH cycle is established during cell cycle progression that involves the dynamic partitioning of cellular GSH between the nuclear and cytosolic compartments. Cell entry into the cycle in early G_1_ is associated with sequestration of GSH into the nucleus (A). At this stage of cell cycle initiation, the nuclear-to-cytosol (n/c) GSH ratio approximates 4. The transient decrease in cytosolic GSH releases feed-back inhibitory effect of GSH on GCL activity and triggers *de novo *GSH synthesis, a process that continues until the feedback control is reestablished. Sequestered intranuclear GSH exists in the reduced form or bound to nuclear proteins, which together changes the GSH/GSSG redox potential (*E*_*h*_) in favor of gene transcription and cell cycle-associated DNA synthesis/replication. Free GSH functions in antioxidant defense that protects against oxidative DNA damage during DNA replication. As yet unclear, free GSH may be regenerated via deglutathiolation of thiolated nuclear proteins, likely catalyzed by Trx1 and/or Grx1. The dissolution of the nuclear envelope in the prometaphase and cytokinesis (cell cycle exit) induces nuclear-to-cytosol GSH export (B) resulting in equal GSH distribution (n/c = 1) in the two compartments in the newly divided cells. Redox-dependent activation of regulatory checkpoints governs cell exit from quiescence (cyclin D1 and associated Cdk4), entry into and progression through cell cycle (cyclin E1-Cdk2, cyclin A-Cdk2 kinase complexes), and final exit from cell cycle (cyclin B1-Cdk1 kinase complex) (blue arrows). Additionally the checkpoints at the G_0_/G_1_ or G_1_-to-S transitions can be bypassed by locally generated ROS (yellow arrows). GSH: glutathione, GSSG: glutathione disulfide, GCL: *γ*-glutamate cysteine ligase; n/c: nuclear-to-cytosol, Trx1: thioredoxin1, Grx1: glutaredoxin 1, and Cdk: cyclin-dependent kinase.

 Cell cycle control is crucial for proper postdamage endothelial repair and growth. The mammalian cell cycle is characterized by a quiescent G_0_ phase of nondividing cells followed by cell entry into the cell cycle at G_1_ and progression through the S, G_2_, and M phases in response to environmental or cellular cues that overcome the biological constraint of a mitotic block [20]. DNA replication takes place during the S phase, and accurate replication commits cell progression into the M phase while aberrant DNA replication induces transient G_2_ arrest that allows for DNA repair [85]. Failure of DNA repair initiates cell cycle withdrawal and permanent senescence. Cell progression through the cell division cycle is governed by regulatory checkpoints controlled by specific serine/threonine cyclin-dependent kinases (CDKs) and their respective cyclin subunits. Specifically, the checkpoints for cell transitions from G_0_/G_1_ to S, late G_1_ to early S, S to G_2_, and G_2_ to M are, respectively, regulated by D-type cyclin D1, D2 and associated with CDK4-6, cyclin E1/CDK2 complex, cyclin A/CDK2 kinase complex, and cyclin B1/CDK1 kinase complex in association with Cdc25 phosphatase [86] (Figure 2).

### 3.2. Glutathione and Cell Cycle Regulation

 The progression of cells through the cell cycle has been linked to dynamic changes in the intracellular redox environment particularly that of the GSH/GSSG redox couple from a more oxidized state prior to cell cycle initiation to a more reduced state throughout cell cycle until cell cycle exit after prometaphase and cytokinesis (Figure 2). Specifically, studies have documented that cell exit from the quiescent stage at G_0_/early G_1_ and entry into cell cycle was characterized by a relatively more oxidizing milieu [87, 88] than that during progression from G_1_ through S to G_2_/M [87, 89]. The redox status of cysteine residues of cell cycle regulatory proteins and their functions were highly sensitive to the intracellular redox environment, which is impacted by cellular production and/or removal of ROS [86]. For example, in actively dividing cells, redox-dependent activation of specific cyclin/CDKs complexes by locally produced ROS allowed for checkpoint bypass at the G_1_ restriction point or at late G_1_ to S transition [7, 14, 90]. Similarly, growth-factor-mediated ROS production and redox regulation of p16, p27, and cyclin D1, which drove terminally differentiated cells into cell cycle [91, 92], governed the reentry of quiescent cells into the cell cycle [91, 92]. E2F, pRB, MAP kinase, Cdc25 phosphatase, and cyclin are other important cell cycle proteins shown to undergo redox changes and/or modifications during cell cycle progression [90, 93–95].

 A role for ROS in mitogenic signaling is underscored by the finding that treatment of serum-starved cells with the thiol antioxidant, N-acetylcysteine (NAC), elicited cell cycle arrest at G_1_, a delay of G_0_ to G_1_ progression that correlated with defective redox control [92]. Interestingly, during exponential growth of cultured mouse embryonic fibroblasts, NAC treatment arrested cells at the G_1_ to S transition but allowed cell transit through the S, G_2_, and M phases [96], indicating that redox control at the early event at G_1_ governed cell progression from G_1_ to S. An increase in MnSOD activity was implicated in NAC-induced inhibition of G_1_ to S entry [90]. Collectively, these studies illustrate the importance of ROS in mitogenic signaling during cell cycle, a redox process that appears to be coordinated through defined cellular mechanisms for ROS generation and elimination. A reduced intracellular redox environment protected genomic DNA from oxidative damage upon breakdown of the nuclear envelope [89] and was therefore essential to enhance DNA synthesis during cell transition from G_1_ to G_2_/M. Early accumulation of soluble thiols at the mitotic spindle was observed during mitosis in sea urchin eggs [97]. Similarly a graduation of low to high GSH content was associated with the transition of Chinese hamster ovary fibroblasts through G_1_ to S to G_2_/M [89], consistent with a well-defined dynamics of redox changes in the intracellular environment during cell cycle.

 Intracellular redox homeostasis is maintained by the thiol/disulfide redox systems of GSH/GSSG, thioredoxin (Trx/TrSS), and cysteine (Cys/CySS). The product of reducing potential and reducing capacity of the redox couples determined the cellular redox environment, which in most cells are largely governed by that of the GSH/GSSG couple [4]. Indeed, the cellular GSH/GSSG redox status provides a good quantitative indicator of the intracellular redox state, often expressed as the redox potential, *E*_*h*_. Under physiological conditions, *E*_*h*_ for GSH/GSSG, as calculated by the Nernst equation, is between −260 mV and −200 mV [15]. Notably, a change in GSH/GSSG *E*_*h*_ from a reduced value of ~260 mV to an oxidized value of −170 mV was associated with phenotypic cell transition from proliferation to growth arrest and apoptosis [15]. As discussed in Section 1.2.1, specificity of redox signaling and independent redox regulation of the functions of single proteins or protein sets are in part attributed to the existence of distinct compartments of GSH within the subcellular organelles.

 Recent evidence suggests that the dynamic cytosol-to-nuclear GSH distribution was a crucial factor in cell cycle progression in that nuclear GSH accumulation provided an intranuclear redox environment that enabled proper regulation of redox signaling events during the various stages of the cell cycle [13]. A novel concept of a nuclear GSH cycle that operated during cell cycle has been proposed [98] as illustrated in Figure 2. According to this hypothesis, GSH was recruited and sequestrated into nucleus in early G_1_ phase, likely through a BcL-2-dependent import mechanism [99]. Increased cytosolic-to-nuclear GSH translocation transiently caused GSH imbalance within the cytosol that initiated *de novo* GSH synthesis, resulting in progressive increases in the total cytosolic GSH pool. Cell transition through G_2_/M and the dissolution of the nuclear envelope during mitosis enabled the reequilibration of the cytosolic and nuclear GSH pools, and this return to a pre-cell cycle nuclear-to-cytosolic GSH ratio of 1 to 1 was maintained in non-proliferating cells at G_0_/G_1_. It was further proposed that it was the transient decrease in cytosolic GSH that promoted early G_1_ signaling. Moreover, the increased GSH presence in the nucleus during the S phase coincided with the activation of DNA replication as evidenced by elevated S-glutathiolation of histones, telomerase, and polyADP ribose polymerase [13, 100]. Additionally, DNA synthesis and replication could be further facilitated by GSH-dependent reorganization of the nuclear matrix and chromatin structure [101]. The details of GSH control of cell cycle checkpoints during endothelial cell proliferation are sketchy and are the subjects of current investigation in our laboratory.

### 3.3. Glutathione Disruption and Implications for Endothelial Growth and Repair

As an organ that is highly dependent on oxidative metabolism for its energy needs, the brain is susceptible to tissue GSH imbalance and oxidative damage mediated by increased formation of free radical species and lipid peroxidation [102, 103]. Given the location of the BBB at the interface between brain parenchyma and systemic blood, the endothelial monolayer is easily exposed to the oxidizing conditions of elevated ROS or RCS associated with various pathological states (Section 4 below). Additionally, an often decreased tissue or systemic GSH level under these diseased states would enhance oxidative damage to the vascular endothelium and the consequent loss of vascular integrity will have important implications for cerebral homeostasis. The enzyme *γ*-glutamyl transpeptidase (*γ*-GT) is regarded as a marker of BBB integrity in the mammalian brain. It is noteworthy that *γ*-GT levels were lowest in the more primitive regions of the brain and highest in the more specialized regions of the brain [104], the reason of which is yet unknown. Importantly, within the brain, the microvesicular fractions exhibited significantly higher *γ*-GT activity than the neuronal or glial fractions, consistent with a microvesicular localization of the enzyme [104]. However, *γ*-GT activity in type I cells (“cobblestone” phenotype) increased 10–12-fold after glial stimulation, indicating a role for type I cells in BBB function as well [105]. Equally notable was the finding that membrane-associated *γ*-GT activity in the endothelium of capillaries was higher than that in larger vessels in the brain, implying that cerebral small vessel endothelial monolayers will likely be more sensitive to fluctuations in the plasma GSH levels in terms of both susceptibility to injury and efficiency of repair. Furthermore, given that *γ*-GT can catalyze the metabolism of not only GSH but also S-nitrosoglutathione (GSNO), cerebral microvascular *γ*-GT function could be pivotal in mediating the bioactivity of GSNO and/or NO (Section 4.2).

 The findings that GSH levels in endothelial cells in culture increased during the lag phase, were elevated during the initial exponential growth phase, and then fell as cells become confluent [106] suggest that systemic GSH interruption would alter endothelial growth. Our recent studies in human microvascular endothelial cells showed that inhibition of GSH synthesis and GSH depletion elicited a delayed S-to-G_2 _transition reflected in a lengthening of the cell cycle S-phase resident time (Busu and Aw, unpublished), in agreement with previous observations [107]. Significantly, cellular GSH depletion was largely confined to the cytosolic pool while the nuclear GSH compartment remained relatively unchanged. Somewhat surprisingly, delayed S-to-G_2_ transition remained evident for 6 h despite the restoration of cytosolic GSH synthetic capacity and near normalization of basal cellular GSH levels (Busu and Aw, unpublished), consistent with a significant time lag between restored cellular redox balance and recovery of normal cell cycle activity. The reason for this temporal dissociation is unclear and is currently under investigation in our laboratory. What is clear, however, is that through perturbing cell cycle events, a disruption in cellular GSH such as that occuring during oxidative or carbonyl stress could delay endothelial proliferation and tissue repair following oxidative damage to the endothelium, a deleterious scenario for brain function in cerebrovascular and neurodegenerative disorders.

## 4. Pathological Implications of Impaired Glutathione in Neurovascular Disease States

### 4.1. Neurovascular Pathology of Diabetes

 Increased BBB permeability has been demonstrated in patients with type II diabetes [108] and in the streptozotocin- (STZ-) induced type I diabetic experimental rat model [109]. Elevated activities of plasma metalloproteinases 2 and 9 were implicated in the loss of tight junctional proteins (occludin, claudin-5, ZO-1, and JAM-1) and BBB failure [110]. Interestingly, the receptor for AGE (RAGE) was upregulated during diabetes [111], suggesting that increased plasma-to-cellular MG uptake and enhanced GSH-dependent intracellular MG catabolism could provide a means to attenuate the elevated systemic MG levels associated with the diabetic state. BBB disruption was notable during diabetic ketoacidosis wherein neurovascular inflammation, accompanying CCL-2 chemokine expression, NF-*κ*B activation, and nitrotyrosine formation were likely contributors to the attenuated BBB integrity and increased barrier permeability [112]. In STZ-induced diabetic rats, BBB function was improved by the administration of growth hormone and insulin [113, 114]. Our recent studies demonstrated that insulin-mediated protection of human microvascular endothelial cells against MG-induced apoptosis was the result of increased intracellular GSH through activation of the insulin-PI3K/Akt/mTOR/Nrf2/GCLc signaling pathway [50, 52].

 It is well known that diabetes is associated with hyperglycemia, elevated oxidative and carbonyl stress, and low tissue and plasma levels of GSH [115–120], conditions that complicate the diabetic state, which would lead to further exacerbation of GSH loss. Thus, mechanisms that promote neurovascular GSH status or those that attenuate oxidative and/or carbonyl stress could preserve endothelial barrier function. A viable approach could involve activation of insulin signaling to maintain cellular GSH balance and support GSH-dependent attenuation of oxidative or carbonyl stress mediated by ROS or MG [25, 51, 52, 121]. Furthermore, increasing GSH protection of redox sensitive thiols of membrane proteins, including those of the AJ or TJ, could preserve the functional integrity of the endothelium. The question of whether acute or chronic GSH therapy would be effective in abrogating systemic hyperglycemia-linked oxidative and carbonyl stress and mitigate diabetes-associated BBB dysfunction remains an open question that warrants further investigation.

### 4.2. Microvascular Dysfunction in Stroke

 Stroke is a cerebrovascular disorder wherein a blood clot or interrupted blood flow to a region of the brain leads to a rapid loss of brain function. Significantly, a lack or delayed flux of oxygen and glucose to the brain will result in neuronal death and brain damage. Clinical studies have shown that subjects at risk for stroke exhibited low tissue GSH levels and decreased GSH-to-GSSG ratio and that the restoration of normal cerebral GSH balance could be as long as 72 h after the ischemic insult [122, 123]. Importantly, acute ischemic stroke was associated with elevated oxidative stress, a major contributor to immediate and delayed ischemic brain injury and changes in the parenchymal GSH redox status [124, 125]. An increase in free radical production during acute cerebral ischemia can arise from multiple sources including stimulation of N-methyl-D-aspartate receptors [126], mitochondrial dysfunction [127], activation of neuronal NO synthase (NOS) [128, 129], autooxidation of catecholamines, and metabolism of free fatty acids [130]. The activation and migration of inflammatory cells, such as neutrophils, further contributed to  O_2_^∙−^ and H_2_O_2_ generation [130].

 The restoration of endothelial integrity after thrombotic or hemorrhagic stroke is crucial to preserving BBB function and neurovascular homeostasis. The proliferation of endothelial cells adjacent to the lesion or injury site is a pivotal step. Given the role of GSH in cell proliferation (Section 3), maintaining cellular GSH balance is therefore essential for postdamage endothelial repair and wound healing. S-nitrosoglutathione (GSNO) is an important physiological metabolite produced by the reaction of NO with GSH [131] that is involved in NO storage and release through the function of *γ*-GT [132]. The affinity of *γ*-GT for GSNO (*K*_*m*_ of 0.4 mM) was comparable to other *γ*-glutamyl substrates [132], suggesting a physiological role for *γ*-GT-in GSNO metabolism. Whether high micromolar concentrations of GSNO are achievable in cells remains uncertain. However, recent studies demonstrated that, at least in plasma, GSNO levels are likely to be higher than previously reported due to the presence of exogenous *γ*-GT [133], further underscoring the significance of the enzyme in modulating GSNO levels and bioactivity.

 Reportedly, GSNO functions in cellular signaling [134, 135] and protection of the central nervous system (CNS) against excitotoxicity, inflammation, and ROS [136, 137]. Notably, GSNO protection against peroxynitrite-induced oxidative stress is severalfold more potent than GSH [138]. GSNO-mediated CNS protection against inflammation appeared to be through suppressing iNOS induction and promoting eNOS expression, and maintaining cerebral blood flow [139]. The anti-inflammatory activity of GSNO in downregulating iNOS was mediated by inhibition of NF-*κ*B activation and decreased expression of ICAM-1 and ED-1. Additionally, the expression of ZO-1 and occludin at endothelial tight junctions was enhanced by GSNO treatment [140]. Unlike conventional NO donors that mediate quick NO release, GSNO elicits slow NO release that was implicated in neurovascular protection against ischemia reperfusion [141]. In addition to *γ*-GT, S-nitrosoglutathione reductase (GSNOR), which catalyzes the reduction of GSNO, has been shown to be an important regulator of the endogenous GSNO levels and NO bioactivity. The pathophysiological role of GSNOR in SNO signaling and NO bioactivity in the regulation of vascular tone is incompletely understood; recent evidence suggests that GSNOR regulates airway SNO levels in cell signaling [142] and protects against nitrosative stress and cancer risk in human lung [143]. This notwithstanding, it remains unclear whether therapeutic strategies involving exogenous GSH and/or NO supplementation during neurovascular inflammatory conditions, such as stroke, would be clinically efficacious in the short term in attenuating the oxidative burden and protecting the BBB, or in the long term in reducing brain edema and tissue damage.

## 5. Summary and Perspective

 The integral function of the microvascular endothelium underpins cerebrovascular homeostasis. ROS- and/or RCS-induced endothelial dysregulation is an underlying concern in barrier failure, and, as such, much research has focused on the use of antioxidants as a strategy to attenuate oxidative or carbonyl stress and restore monolayer function. The finding that GSH, a major cellular antioxidant, is able to afford cytoprotection supports the notion that antioxidant therapy is important in endothelial barrier preservation. In past years, more recent conceptual advances in redox cell biology have uncovered a fundamental role of GSH in signal transduction and redox signaling in cellular functions. Moreover, the finding that distinct pools of GSH exist in subcellular organelles that allow for independent redox regulation has revolutionized our thinking of GSH-dependent redox mechanisms in controlling metabolic processes. One such biological process is that of cell proliferation. In the context of enhanced endothelial proliferation and self-repair surrounding lesion sites in response to systemic cues, for example, growth factors, little is known of a role for GSH. The dynamics of cytosol-to-nuclear GSH distribution appears to be pivotal in governing cell cycle responses. The notion that cell proliferation and growth can be a relevant biological process for monolayer repair/restitution following endothelial injury in much the same way as epithelial cell restitution/proliferation restores postinjured epithelium suggests exciting new avenues for future research in endothelial biology. Importantly, an understanding of GSH control of endothelial cell proliferative potential under different oxidizing conditions and plasma GSH levels will expand our perspective for future development of therapeutic strategies. Targeting endothelial restoration after oxidative insult and tissue damage is likely to be clinically relevant to the neurovascular disorders of diabetes and stroke and additionally could have broader implications for neurodegenerative and neurological disorders as well.

## Abbreviations

AGE: advanced glycated end product
AJ: Adherens junctions
AP-1: Activator protein-1
AP-2: Activator protein-2
ARE: Antioxidant response element
BBB: Blood-brain barrier
CDK: Cyclin-dependent kinases
CNS: Central nervous system
Cys: Cysteine
CySS: Cystine
*E_h_*: Redox potential
eNOS: Endothelial nitric oxide synthase
EpRE: Electrophile responsive element
GCL: *γ*-glutamyl cysteine ligase
GCLc: GCL catalytic subunit
GCLm: GCL modifier subunit
GRP: Glucose-related protein
Grx: Glutaredoxin
GSH: Glutathione
GSNO: S-nitrosoglutathione
GSNOR: S-nitrosoglutathione reductase
GSSG: Glutathione disulfide
H_2_O_2_: Hydrogen peroxide
Hsp27: Heat shock protein 27
Hsp60: Heat shock protein 60
IP3: Inositol-1,4,5-trisphosphate
IP3R: IP3 receptor
MG: Methylglyoxal
NAC: N-acetylcysteine
NF-*κ*B: Nuclear factor kappa B
NO: Nitric oxide
NOS: NO synthase
Nrf2: Nuclear factor E2-related factor 2
O_2_^∙−^: Superoxide anion radical
RCS: Reactive carbonyl species
ROS: Reactive oxygen species
STZ: Streptozotocin
TJ: Tight junctions
Trx: Thioredoxin
ZO: Zonula occluding protein
*β*-NF: *β*-naphthoflavone.
